# Parochial cooperation in wild chimpanzees: a model to explain the evolution of parochial altruism

**DOI:** 10.1098/rstb.2021.0149

**Published:** 2022-05-23

**Authors:** Sylvain R. T. Lemoine, Liran Samuni, Catherine Crockford, Roman M. Wittig

**Affiliations:** ^1^ Taï Chimpanzee Project, Centre Suisse de Recherches Scientifiques, Abidjan, Côte d'Ivoire; ^2^ Max Planck Institute for Evolutionary Anthropology, Leipzig, Germany; ^3^ Department of Archaeology, University of Cambridge, Cambridge, UK; ^4^ Department of Human Evolutionary Biology, Harvard University, Cambridge, MA, USA; ^5^ Institute of Cognitive Sciences Marc Jeannerod, UMR 5229, CNRS/University of Lyon, Bron, France

**Keywords:** human evolution, primates, social ties, oxytocin, in-group out-group, parochial cooperation model

## Abstract

Parochial altruism, taking individual costs to benefit the in-group and harm the out-group, has been proposed as one of the mechanisms underlying the human ability of large-scale cooperation. How parochial altruism has evolved remains unclear. In this review paper, we formulate a parochial cooperation model in small-scale groups and examine the model in wild chimpanzees. As suggested for human parochial altruism, we review evidence that the oxytocinergic system and in-group cooperation and cohesion during out-group threat are integral parts of chimpanzee collective action during intergroup competition. We expand this model by suggesting that chimpanzee parochial cooperation is supported by the social structure of chimpanzee groups which enables repeated interaction history and established social ties between co-operators. We discuss in detail the role of the oxytocinergic system in supporting parochial cooperation, a pathway that appears integral already in chimpanzees. The reviewed evidence suggests that prerequisites of human parochial altruism were probably present in the last common ancestor between *Pan* and *Homo*.

This article is part of the theme issue ‘Intergroup conflict across taxa’.

## 1. Introduction

The parochial altruism hypothesis [1] has been proposed to explain the human tendency for in-group favouritism and out-group hostility, suggesting that out-group conflicts drive in-group cohesion and cooperation [1,2]. The hypothesis postulates [1] that groups with more individuals to favour the in-group over the out-group (parochialism) are more cooperative during an out-group conflict (i.e. those that confer benefits on others at an immediate cost to self). Therefore, they are likely to triumph over out-groups and gain substantial benefits, thereby reinforcing the adaptive value of the parochial cooperative phenotype. However, the importance of out-group conflict as an evolutionary driver of human in-group cooperation [3,4] is debated (reviewed in [5]). Instead, competing theories suggest the role of increased collaborative breeding [6] and foraging needs [7] as alternative selective pressures shaping human cooperation capacities. To reveal the evolutionary foundations of human adaptations, we can rely on evidence from closely related species as windows into our past. To explore the role of parochial altruism in hominin evolution, we review evidence on the impact of intergroup competition on both individual fitness and in-group cooperation in one of our closest living relatives, chimpanzees (*Pan troglodytes*), a species with intense competition between groups. Given that chimpanzees are neither cooperative breeders nor obligatory collaborative foragers allows us to explore the role of intergroup competition on cooperation independently of these alternative hypotheses.

Evidence suggest that human parochialism (in-group favouritism) emerges early during development [8,9], suggesting a biological basis without the influence of cultural norms. Also, in-group solidarity tends to increase in response to out-group conflict [10,11], suggesting a causal link between out-group threat and in-group cohesion. Further, the mobilization of a deeply rooted neurohormonal pathway involving the oxytocinergic system has been proposed as a proximate physiological mechanism regulating in-group cooperation against an out-group threat [12]. Despite the potential impact of out-group hostility on the evolution of human cooperation capacities, the evolutionary trajectories of parochial altruism have received little attention so far. Investigating the evolutionary roots of in-group cooperation and out-group hostility are crucial if we wish to understand the selection pressures which have shaped human parochial altruism. In this review, we bring together evidence suggesting that key elements of human parochial altruism are probably shared with one of our closest living relatives, chimpanzees. We review decades of research on wild chimpanzees across their biogeographical range, adding insights from recent advances using various methodological approaches, assessing links between out-group conflicts, group-level cooperation and physiological mechanisms sustaining group-level cooperation and out-group aggression in chimpanzees.

## 2. In-group cooperation in the face of out-group threat: a cross species perspective

For territorial, group-living species, out-group conflicts may have deleterious effects on survival and reproduction [13]. Evidence across taxa, including birds and mammals, demonstrate an immediate increase of in-group cohesion and affiliation following out-group threat [14], pointing towards the link between out-group conflicts and in-group favouritism. In some of these species, such as green wood hoopoes (*Phoeniculus purpureus*), in-group cooperation increases before and after out-group conflicts among individuals who are more likely to suffer fitness costs owing to out-group conflicts [15,16], therefore suggesting an individual-based fitness cost/benefit response to the threat. Cooperative acts among many group members (hereafter group-level cooperation) also occur before and during out-group conflicts in birds [15], social carnivores [17–19] and primates [20]. In-group members probably differ in the benefits they may gain and the costs they may suffer owing to out-group conflicts, and individual cost/benefit trade-off seems to explain variation in individual participation, where individuals who have more to lose in case of defeat participate more [20–22]. However, while there are probable inter-individual differences in the costs and benefits of out-group conflict participation, the participation of all individuals is associated with a large uncertainty with regard to the immediate costs and long-term returns. While human parochial altruism implies self-sacrificial actions that benefit the group, in non-human taxa, participation motivations in out-group conflicts are probably largely driven by mutual benefits [23] or even selfish interests. Nonetheless, whether human participation in out-group conflict is purely altruistic can be questioned. Owing to the lack of evidence for self-sacrificial behaviour during out-group conflict in non-humans, we hereafter use the term ‘parochial cooperation’ instead of altruism in our review on chimpanzees.

Joint territorial defence in most animal species occurs among kin and can therefore be explained by inclusive fitness mechanisms. In-group cooperation amongst non-kin, however, is more difficult to explain, especially when benefits are not immediately gained or are gained independently of contribution into the cooperative act. In chimpanzees, similar to humans, unrelated adults frequently cooperate [24], both at the dyadic [25,26] and the group level [27,28]. Group-level cooperation in chimpanzees is observed for example in collective hunting events [29–31] and territorial defence [24,32,33]. Since in these contexts the cooperative act often occurs among non-kin, cooperation might be unstable. For example, when the cooperative act allows us to secure a public good (i.e. territory), then access to benefits of cooperation is gained by all in-group individuals whether or not they invested in the costly act (i.e. territorial defence). Therefore, for the single individual, defection during hostile intergroup encounters (IGEs) may be a more profitable strategy, leading to a collective action problem.

In humans, the mechanisms sustaining non-kin cooperation have been under scrutiny since Darwin [34] and led to various hypotheses (reviewed in [35,36]). Within these models, intergroup competition has been proposed as an important evolutionary selective pressure, leading to the parochial altruism hypothesis [1]. First, experimental approaches in humans revealed that preferences for in-group at the expense of the out-group in a competitive context appear early in life [8,9,37], suggesting a developmental and potentially evolutionary basis for human parochial altruism. Second, in-group favouritism emerges spontaneously in naturally occurring groups, coupled with out-group hate in between-group competitive contexts [38], suggesting that out-group hostility emerges when facing an out-group threat. Third, advances in neuroscience and neurophysiology underscore the role of the neuropeptide oxytocin in human parochial altruism by promoting both in-group cooperation and solidarity in humans as well as defensive aggression towards competing out-groups [12]. The same mechanism was suggested to also underlie chimpanzee in-group cooperation during out-group conflict [39].

However, in humans, alternative selective pressures have been suggested to select and maintain non-kin cooperation, such as joint hunting and gathering [7] and cooperative breeding [40]. Chimpanzees are not cooperative breeders, but they do undertake group-level cooperative actions when hunting on animal prey [29,30,41], although these are probably unnecessary for their survival. In humans, it has also been argued that in-group favouritism can emerge in the absence of out-group threat [5,42], unconditional of out-group discrimination, where in-group membership indicates a higher probability of reciprocal interactions. While this group heuristic approach involves the absence of out-group threat, in chimpanzees and most likely in most social vertebrates, other groups *de facto* constitute a threat in an intergroup competition context. Consequently, reviewing the impact of out-group hostility on in-group cooperation can help contextualize the relevance of out-group conflicts as a potential selective pressure in hominin evolution.

Given the evolutionary proximity between humans and chimpanzees, chimpanzee collective action during out-group conflict and the deeply evolutionary rooted oxytocinergic system [43], we propose to investigate whether prerequisites of parochial cooperation are at play in chimpanzees. Given the documented relationship between out-group conflicts and in-group cooperation in other animal species, we discuss the possibilities that parochial cooperation corresponds to analogous adaptations. We also incorporate an essential puzzle piece in this phylogenetic comparison between chimpanzees and humans, by discussing evidence for parochial cooperation in bonobos (*Pan paniscus*), our other closest living relative.

### The parochial cooperation model for non-human animals

**Figure 1.**
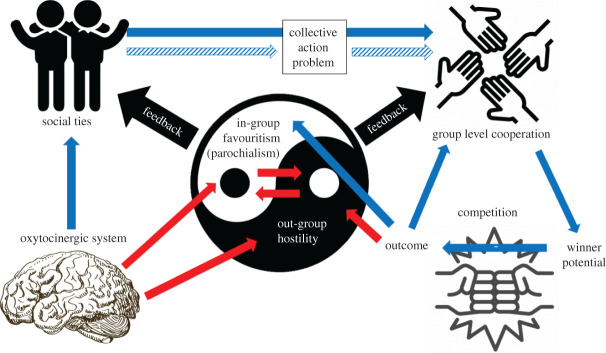
The parochial cooperation model. Variables and pathways involved in small-scale group cooperation of non-human animals in an in-group out-group context. Blue arrows depict pathways with empirical support, while red arrows depict pathways empirically untested. Solid arrows depict increasing effects while patterned arrows correspond to decreasing effects. Black arrows suggest potential feedback loops in the model.

In contrast with large-scale cooperative systems between unrelated and unfamiliar individuals observed in humans and human ancestors [44,45], we refer to a parochial small-scale group cooperation model for non-human animals (figure 1), hereafter *parochial cooperation model* (PCM), where all individuals in the in-group are familiar to each other. In this model, social ties, constructed through repeated interactions and promoted by the oxytocinergic system, constitute a cement by which non-related individuals maintain group-level cooperation. In the PCM, social ties counter-act collective action problems which leads to better group-level cooperation [32]. Group-level cooperation then leads to a better competitive ability/higher winning potential in intergroup conflicts [46], thereby reducing the negative effects of out-group conflicts on reproductive success (outcome) [47]. Strong intergroup competition reinforces in-group favouritism and cohesion, strengthening the cementing power of the social ties and reinforces out-group hostility through the mobilization of the oxytocinergic system [48].

We use chimpanzees to examine some pathways of this model, by reviewing patterns of within-group cooperation during intergroup conflicts (§3), the evolutionary significance of intergroup conflicts and their selective potential on group-level non-kin cooperation (§4), and the importance of the oxytocinergic system on social bonding, cooperative actions and out-group hostility (§5). We also review similarities and differences in non-kin cooperation, out-group hostility and intergroup competition between chimpanzees and bonobos (§6), to better assess the potential evolutionary roots of human parochial altruism.

## 3. Chimpanzee out-group conflicts: ubiquity, imbalance of power and cooperation

Competition for resources among neighbouring groups is common in many group-living animals [13]. Intergroup competition and out-group aggression are associated with territoriality, where individuals from a given group defend a spatial area against the intrusion of neighbouring groups [49,50]. One feature of intergroup competition is the occurrence of regular hostile IGEs. The degree of violence and danger inherent to IGEs is variable across taxa, spanning from ritualized displays, vocal exchanges, to direct physical contacts including chases, fights and killings [13]. Thus, the highly variable potential costs of IGEs across taxa suggest that out-group conflicts do not pose the same selective pressure for all species. Alongside with some social carnivores [17,51,52], chimpanzees fall into the extremity of intensity of out-group hostility and violence observed in non-human animals, and together with their close relatedness with humans they are often used as a model species to examine the evolutionary origins of in-group cooperation and parochialism. This section reviews the intensity of territorial behaviour across chimpanzee populations. It reveals that intergroup hostility is nearly ubiquitous and that cooperation is a fundamental part of intergroup interactions (right side of figure 1), being elicited when detecting neighbours, being adopted to reduce risks during territorial behaviour and being determinant in out-group conflict outcomes. However, while hostile and violent out-group conflicts in chimpanzees are nearly ubiquitous, the intensity and costs of these conflicts vary between populations [53], which may be owing to between-population differences in socio-ecological conditions and resulting resource availability. We explore these differences, which can give insights into variation in parochialism observed in human populations [54], and argue that the variation in social cohesion across populations explains the variability in the intensity of out-group conflicts in wild chimpanzees.

### (a) Ubiquity of hostile intergroup interactions

Chimpanzees are male philopatric and live in multi-male multi-female communities composed of genetically related and unrelated individuals [27,28]. Not all individuals in a chimpanzee community permanently associate with one another but instead they split during the day into sub-groups variable in size, duration and composition (i.e. fission–fusion dynamics [55]). Given their fission–fusion social dynamics, not all in-group members are available as interaction partners at any given moment, rendering the social cohesion variable. Neighbouring chimpanzee communities share territory overlaps constituting high risk areas [56,57]. Territoriality in wild chimpanzees is expressed in several forms: IGEs are regular and mostly constitute distant vocal exchanges [32], but at times they also include direct IGEs with visual and/or physical contact, during which individuals chase and attack the out-group [27,28,58]. IGEs are hostile and stressful events [59–61] and can escalate into lethal aggression [53]. Chimpanzee territoriality is also expressed by in-group patrols of territory borders, where chimpanzee typical fission–fusion patterns change to become more cohesive [39] as they travel silently towards border areas [33,62].

**Figure 2.**
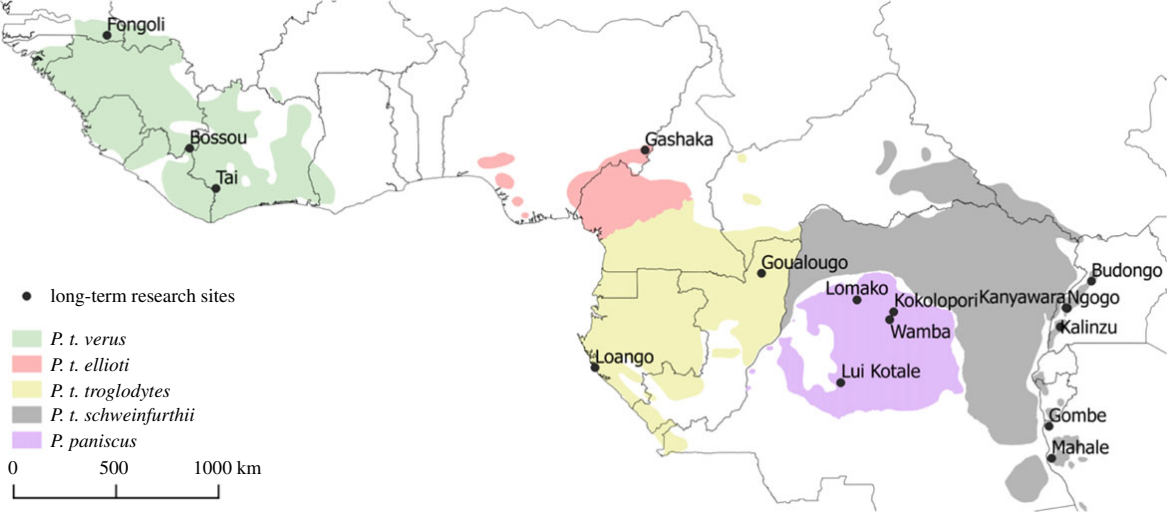
Distribution of chimpanzees (*Pan troglodytes*) and bonobos (*Pan paniscus*) across Africa. Chimpanzees divided in four subspecies (*verus*, *eliotti*, *troglodytes* and *schweinfruthii*) marked in different colours. Long-term study sites are marked for better understanding of the geographical distribution.

Despite variation in intensity of out-group aggression, chimpanzee territorial behaviour and strategies are remarkably similar across well-studied populations and subspecies. In most studied populations, border patrols, hostile IGEs and intergroup killings have been reported (figure 2; eastern chimpanzee *Pan troglodytes schweinfurthii* populations: Budongo [53,63,64], Gombe [27,65–68], Kalinzu [53,69], Kibale (Kanyawara [53,70,71], Ngogo [72–76]), Mahale [77–79]; central chimpanzee *Pan troglodytes troglodytes* populations: Loango [80,81], Goualogou [53]; western chimpanzee *Pan troglodytes verus* populations: Taï [82]). To date, no information is available for Nigeria-Cameroon chimpanzees (*Pan troglodytes ellioti*). Victims of intergroup killings were mostly adult males and infants, but also included a few adult females (reviewed in [53]). So far, no intergroup killing has been reported from two western chimpanzee communities: Bossou, Guinea, and Fongoli, Senegal. In Bossou, a community surrounded by human settlements [83,84], no intergroup interactions have been witnessed and border patrols are uncommon [83,85]. In Fongoli, adult males do not show patterns of boundary patrol [86] and no intergroup killing has so far been observed. This absence could be owing to the very large home-range occupied by the Fongoli chimpanzees [87] and the low population density [88] possibly reducing the chances to encounter neighbours. Apart from these exceptions, it is largely accepted that hostile intergroup relationships in chimpanzees are ubiquitous and can carry substantial costs.

### (b) Imbalance of power and cooperation

The occurrence of intergroup killings and escalation of intergroup violence in wild chimpanzees typically depend on the imbalance of power between opponents [70]. Imbalance of power can be unpredictable, owing to the fission–fusion association dynamics of chimpanzee groups, where small parties can be outnumbered by larger neighbouring parties [70]. Numerical assessment is thus crucial to decide whether or not engaging into a conflict may be beneficial. Chimpanzee capability for numerical assessment during intergroup interactions has been assessed using playback experiments in both wild eastern and western chimpanzees [89–91]. An audio playback of long-distance pant hoot vocalizations of an out-group male elicited approach responses only by parties composed by at least three adult males [90]. In combination with mathematical models, playback experiments suggest that contest decisions are taken when a party outnumbers the out-group by a factor of 1.5 [89]. In Taï and Ngogo, patrolling parties that approached intruders tend to be larger and to contain more adult males than patrolling parties that retreated from the intruders' location [28,33], and generally parties travelling in the periphery of the territory are large [57]. Further in Taï, in-group numerical strength impacts IGE participation decisions of both males and females [32]. Other playback experiments [91], where different categories of potential intruders were displayed (familiar neighbours, unknown neighbours or in-group members) revealed a capacity to recognize individuals from their pant hoots, with a more cautious response when unfamiliar intruders were heard than when familiar neighbours or in-group individuals were heard. When at least three adult males were present in the party, the in-group responded to the out-group playback calls by producing long-distance vocalizations, but otherwise remained silent. Knowing that these playbacks simulated the intrusion of a single individual, these experiments corroborate the 1.5 numerical assessment ratio found elsewhere [89], and conform with theoretical models of asymmetries in animal conflicts [92].

Aside from numerical assessment capacity, playback experiments demonstrated collective responses to simulated intrusions, characterized by joined chorus loud vocalizations [90], coordinated approaches to the speaker [90] and patrolling behaviour [91]. Pant hoot chorus in wild chimpanzees may reflect social bonds and predict engagement in affiliative behaviour [93]. Patrolling behaviour, with reinforced spatial cohesion [39] and coordinated collective movements are striking examples of group-level cooperation in chimpanzees [94]. The ability to cooperate as a group is thus an important territorial response in chimpanzees, with cooperation being elicited as a response to a potential direct or indirect out-group threat. In addition, cooperative response to out-group threat is revealed by the formation of in-group alliances to attack and severely injure out-group members. Coalitionary aggressions against the out-group are most ‘profitable’ in times of power imbalance in favour of one's own group, by reducing the costs of conflict participation and increasing the chances of accessing potential benefits [70]. Thus, cooperation is key in maximizing the chances of beneficial outcomes of out-group conflicts, while the escalation of conflicts depends on the imbalance of power between opponents.

### (c) Cross-population differences in rates of intergroup killings

Studied populations of eastern, central and western chimpanzees show similar patterns of territoriality and border defence, hostility towards neighbours and coalitionary aggression against out-group individuals. Despite ubiquitous characteristics of chimpanzee intergroup relations and territoriality, the rates of intergroup killings vary across chimpanzee populations [53]. Chimpanzee lethal aggression, within and between groups, typically increases with more adult males and higher population density [53]. Rates of intergroup lethal aggression are high in eastern populations as compared to western chimpanzees. Some eastern chimpanzee populations live in high population density and groups are composed of many adult males, whereas in some western chimpanzee populations more recent demographic changes have led to a low population density and typically fewer adult males per community (Taï and Bossou). Nonetheless, even though population density and community sizes in Taï were higher in the past [95], rates of lethal killings in this population remained consistently low.

Between-population differences in lethal aggression may also be owing to differences in ecological conditions, where higher and more predictable food availability and distribution are associated with reduced intragroup feeding competition, allowing for larger mixed-sex parties and thus more equal balance of power during IGEs [96]. This hypothesis was also suggested for the absence of intergroup lethal raiding in bonobos [70]. However, systematic comparative methodological approaches in assessing food availability, distribution and predictability across sites would be necessary to test this hypothesis. Variation in population history [97,98] may also explain between-population differences in population density, which in turn may relate to between-population differences in rates of lethal aggression. We should also note that the level of habituation to human observers of communities that are neighbouring to the studied habituated chimpanzee communities may also explain some of the observed differences in rates of lethal interactions across sites. It is expected that habituated communities with non-habituated neighbours may have a competitive advantage during IGEs, as the fear of humans may disrupt cooperative response of non-habituated communities. Greater attention to the impact of differences in habituation status on rates of in intergroup killings is warranted.

### (d) Social cohesion reinforces group-level cooperation and reduces the likelihood of intergroup killings

However, it is likely that population density and the number of males do not directly influence intergroup killing rates, but instead impact the social dynamics of groups (e.g. within-group competition and cohesion) which in turn influence the chance for lethal IGEs. For example, within-group cohesion (larger association parties and lower tendency of the group to fission) is expected to increase with a reduction in the number of adult group members [99], and stronger within-group cohesion reduces imbalance of power opportunities and hence conflict escalation [39,82]. In turn, social cohesion and bonding promote cooperation [26], including group-level cooperative territorial behaviour (coalitionary attacks and participation in border patrols) [32]. Thus, between-population differences in intergroup lethality could be linked to differences in social dynamics that may impact group-level cooperation. In comparison to many eastern chimpanzee populations [100,101], western chimpanzees are considered more gregarious, and show lower rates of fission–fusion and larger within-group home-range overlaps among males and females [102–105], whereas in some eastern chimpanzee populations, females occupy smaller home-ranges and are considered less gregarious than males [106]. In addition, while both eastern and western chimpanzee males form social bonds with unrelated individuals [25,107], female bonding patterns are more variable across populations [108,109]. Between-population differences in social dynamics and grouping patterns probably arise owing to variation in within-group competition that is related to community size. However, an alternative, non-mutually exclusive explanation that may cause chimpanzees to maintain large parties is predation pressure, with higher reported rates of leopard predation in western than in eastern chimpanzees [110,111]. While reduced leopard density in many eastern chimpanzee sites is likely to be a recent phenomenon, the fission–fusion social dynamics of chimpanzees affords them great flexibility to respond to environmental fluctuations, including predation risk.

Differences across populations in social cohesion are likely to directly impact patterns of territorial defence and cooperation. For example, female participation to IGEs in some western chimpanzees is common compared to eastern populations [82], possibly owing to higher group gregariousness and cohesion in the former. Whereas sexually mature males are the main patrol participants across populations [28,32,33,62,81,112], strong population differences exist in the propensity of female participation in border patrols [28] being greater in western [28,39] compared with eastern populations [33]. It remains unclear whether relatively low rates of intergroup killing in western chimpanzees encourage improved participation of both males and females or whether increased group cohesion in the first place results in lower killings. Nevertheless, existing variation in social grouping patterns across chimpanzee populations emphasizes the role of social cohesion in reducing the potential costs of territorial defence.

These cross-population comparisons of IGEs and border patrol characteristics confirm that chimpanzee intergroup competition, in-group cooperation and social cohesion are intimately linked (figure 1): social cohesion and bonding promote cooperative territorial behaviour (coalitionary attacks, patrolling behaviour and female territorial participation), which enables a reduction of the risks and increases the odds of winning a conflict. Variation in sociality patterns then in turn impacts the odds of imbalance of power and thus the observed variation in intensity and outcomes of intergroup competition across populations. Out-group hostility occurs in all chimpanzee populations and, even if rates of lethal aggressions vary, intergroup competition in chimpanzees is associated with a high uncertainty of risks and potential benefits. Nonetheless, individuals are probably able to mitigate potential costs and uncertainty by acting together as a cohesive unit.

## 4. Cost and benefits of chimpanzee out-group conflicts

To further unravel the evolutionary links between out-group aggression and cooperation, one needs to consider the cost-to-benefit ratio of out-group conflicts and cooperative territorial behaviour, both at a proximate and ultimate level. This section thus reviews the costs and benefits inherent to IGEs and border patrols in wild chimpanzee populations and proposes a potential selective pathway of intergroup competition on fitness and cooperation (figure 1).

### (a) Selective pathway of intergroup conflicts

Chimpanzee out-group conflicts can incur substantial direct costs, in the form of injuries and death. Death rates owing to chimpanzee intergroup aggression, calculated from nine communities in five populations, were 69–287 per 100 000 individuals per year, comparable with intergroup killing rates in subsistence hunter–gatherer and farmer human populations [71]. Since these calculations, done in 2006, more cases of intergroup killings among the same and additional chimpanzee populations and communities have been reported [58]. In Ngogo, a large eastern chimpanzee community, from Kibale, Uganda, mortality rates of a neighbouring community owing to out-group aggression from the Ngogo community exceed rates from horticultural and hunter–gatherer populations by factors of 1.5–17 altogether [75]. However, given that exact demography of that neighbouring community was unknown, those numbers may be overestimated. Rates of non-lethal injuries following intergroup aggression have not yet been compiled, but the fact that only about a quarter of IGEs involve physical contact [27,32,47] shows that engaging in risky physical fights during IGEs tends to be avoided.

Other types of costs are also incurred by out-group conflicts. In Ngogo, a series of fatal attacks over 10 years on a neighbouring community led to a significant territorial expansion by the Ngogo community [75]. Costs of out-group conflicts in term of territory loss had beforehand been documented in other populations: after a series of lethal attacks, the Gombe Kasekela community took over the territory of their neighbouring Kahama community [27]; in Mahale, the M community annexed the territory of the K community after all males from the K community disappeared [79]. Territorial expansions after intergroup conflicts emphasize that neighbouring communities of chimpanzees compete over space. Territorial expansions increase feeding opportunities, reduce within-group competition, and therefore offer reproductive benefits. This relationship between intergroup competition, the reduction in within-group competition owing to territory size increase, and the associated fitness benefits, was formulated by the intergroup dominance hypothesis [113]. This model postulates that intergroup competition results in a form of group hierarchy, in which the most dominant groups benefit from larger territories, which mediate fitness advantages through access to resources. For example, in eastern chimpanzees, reproductive advantages of living in large territories were found, including increased body mass [114], shorter inter-birth intervals and higher infant survival, measures of improved reproductive success [115]. In the Taï population, where larger groups benefit from larger territories while territorial expansion is mostly determined by the number of in-group adult males [46], shorter inter-birth intervals were found for females living in larger territories [116], and for females living in communities with more adult males [47]. Also in Taï, IGEs had prolonged effects on reproductive success: inter-birth intervals were longer during periods of high neighbour pressure and high neighbour pressure during pregnancy was associated with reduced infant survival [47]. These effects may be owing to the stress incurred by repeated intrusions and IGEs, which have shown to be associated with increased hypothalamic-pituitary-adrenal axis activity during IGEs compared with controls across western and eastern chimpanzee populations [59–61].

Indirect costs of intergroup conflicts through competition for space, such as territory loss, and potential physiological effects of out-group conflicts on reproductive success suggest that intergroup conflicts constitute an important selective pressure. Border patrol engagement is probably also associated with immediate costs. Conducting border patrols incurs potential feeding and reproductive costs, as non-participating males may be able to gain mating opportunities in the absence of sexual competitors, and as feeding time is reduced while energetic expenditure owing to longer traveling distances is increased while patrolling [117]. Patrolling behaviour mostly occurs in the border of territories, areas where the risks to encounter hostile neighbours and thus suffer injury are high [57], and IGEs are often preceded by border patrols [94]. However, in the long term, the immediate costs associated with border patrols may be negligible if border patrols allow individuals to access delayed benefits, such as securing space and feeding grounds and increasing the safety of group members. Furthermore, the chances of suffering injuries owing to encountering neighbours during patrols can be mitigated via strength in numbers [32,70]. However, while the delayed outcomes of border patrols and IGEs may be overall more beneficial than costly, participants in these acts still suffer immediate costs with no guarantee that they will see a return.

### (b) Fitness benefits of intergroup encounters: mate attraction and territorial expansion

An additional benefit of intergroup conflict success and territorial expansion may be through mate attraction. When the Gombe Kasekela community took over the territory of the Kahama community, females from Kahama integrated into the Kasekela community [118]. Similarly, female integration occurred in Mahale after the M community expanded over the territory of the neighbouring K community [79]. These examples of territorial expansion, involving lethal aggression of male competitors, took place in populations presenting a male-bonded community model [103], in which male home-ranges are larger than those of females, who spend most of their time in smaller and distinct core areas [106]. Thus, territorial expansion may not necessarily attract neighbouring females, but rather lead to the annexation of female core areas. A similar phenomenon of neighbouring females home-range annexation was also observed in the bisexually-bonded community in Taï in which both sexes share the same home-range, where a recent wave of female immigration into the south group was observed, leading to an expansion of the South group territory into the area thought to have been these females' former home-range [104]. This case of territorial expansion and female integration was not the result of intergroup competition but was rather owing to the disappearance of a neighbouring community (presumably including all adult males) probably because of poaching (Taï Chimpanzee Project 2019, unpublished data). In the Gombe Kasekela community, territory increase was not associated with an increase in the number of adult females [115], and a reduction in territory size did not lead to the dispersal of females but rather to a decrease in female home-ranges [101] suggesting that territorial expansion does not always lead to female attraction. Also, annexation of neighbouring female home-ranges may occur only in certain conditions, when the remaining individuals no longer participate in territorial maintenance. North Group in Taï had to survive for 3 years without any adult male [95]. The remaining females were not annexed or dispersed but instead ranged together in a smaller part of their former territory and continued to maintain their territory by engaging in border patrols and IGEs (Taï Chimpanzee Project, R. M. Wittig, C. Crockford 2011, unpublished data). In summary, evidence for reproductive success benefits associated with intergroup aggression are mostly based on territorial expansion offering larger feeding grounds to in-group members and diminishing the within-group competition pressure.

### (c) Benefits of border patrols

For the case of IGEs, cooperation increases the odds of winning conflicts, which probably reinforces cooperative phenotypes. The impact of territorial defence on fitness outcomes has also been suggested for chimpanzee patrolling behaviour [112,119]. A longitudinal study in the Ngogo community [112] revealed that males which had more to gain since they had many offspring in the group were more likely to patrol. Nevertheless, an individual cost/benefit approach did not explain all patterns of patrolling behaviour. Authors suggested that group augmentation theory may explain some aspects of patrolling behaviour in this population, in the sense that individuals bear the short-term costs of collective action even if they have little to gain immediately, because territorial defence has a long-term positive impact on access to resources, group size and reproductive success [112]. However, these potential long-term benefits of territorial cooperative maintenance were not directly measured. Nevertheless, analyses of patrolling efforts in the same community [120] showed that, in addition to individual-level attributes (number of patrols conducted by each individual), aggregate-level traits (aggregate number of participating males) had significant effects on the relative fitness of individuals, suggesting fitness benefits of collective action. However, since most of these border patrols led to IGEs, potential fitness outcomes may result from IGE outcomes. These findings, which remain to be replicated in other study sites and so to be confirmed, suggest that participation to border patrols involves not only immediate but also long-term fitness benefits associated with securing and potentially expanding a territory, thereby reducing within-group feeding competition and improving group members reproductive success. Finally, patrolling behaviour may also benefit the entire group by simply securing the territory against neighbouring intrusions even if, so far, such a relationship has not been investigated. These cited studies, however, addressed fitness benefits of males [119], and, so far, little is known about the effect of border patrols on female reproductive success. However, following the logic presented earlier, securing a territory and potentially expanding it get reflected in positive fitness outcomes for females, which can be guaranteed by regular border patrols. Finally, fitness outcomes for females participating in border patrols remain unknown and would benefit from further investigations.

The cost/benefit approach of intergroup competition in several populations of wild chimpanzees and their effects on reproductive success (see also [121]) suggest that out-group conflicts in this taxon constitute a relevant selective pressure, potentially favouring the emergence and maintenance of cooperation among non-related individuals, the maintenance of strong social ties, in-group favouritism and hostility towards out-group members.

## 5. Expanding the link between intergroup competition, in-group cooperation and social ties

This section aims at expanding the evolutionary link between out-group conflicts, in-group cooperation and social ties. We review the theory behind collective action problems and discuss whether chimpanzees can overcome it. We explore the potential role of social ties and the oxytocinergic system in promoting collective action and cooperation (top part figure 1).

### (a) Collective action problems

Collective territorial defence allows groups to secure a public good (i.e. territory), a resource accessible to all in-group members independent of their contribution to the collective act. As potential benefits of territorial defence are accrued in all group members and because territorial acts are associated with immediate costs, a profitable strategy for individuals can be to withhold from participating in territorial maintenance, thereby leading to a collective action problem.

Evidence demonstrates how the collective action problem materializes across primate species. Comparative analyses indicate that group size is positively correlated with between-group home-range overlap in primates [122], suggesting that territory defensibility is potentially impaired for larger groups. Additional comparative studies show that, among primates, intergroup competition is less intense (measured by rates of aggressive IGEs) in species facing collective action problems [123], illustrated by the reduction of territorial behaviour and advertisement in these species. However, conclusions from these studies also suggest that collective action problems vary across primate species. In species where the dominant sex is philopatric and where effective territorial defence is critical for reproductive success and survival, groups can overcome the collective action problem [122]. Chimpanzees fit well within this model, as males are both the philopatric and dominant sex. Further, the intergroup interactions of chimpanzees are particularly hostile and violent [124], and can even lead to the complete loss and dissemination of a group [27,75,125]. As higher costs of territorial defence in chimpanzees are assumed to elicit a stronger collective response by in-group individuals [126], in-group cooperation during out-group conflicts is assumed to be more pronounced in this species. The highly structured and collective border patrols and large coalitionary attacks observed in chimpanzees indeed suggest strong links between group-level cooperation and out-group threat, but the proximate mechanisms that sustain the collective act when benefits are uncertain are less known.

### (b) Privatizing the collective action problem: the role of impact individuals

A proximate mechanism that may allow groups to overcome the collective action problem can result from the influence of a few impact individuals [127,128]—individuals who have more to gain from proactive engagement and thus are more willing to suffer the costs of initiating the act. Once the risky act has already been initiated, the additional participation of other individuals is associated with reduced costs—presumably increasing collective engagement. For instance, in wild chimpanzees, males’ participation to border patrols is heterogenous [33,94,112], collective prey hunting can be influenced by the presence of impact hunters [29,129], and some individuals are the first to attack the out-group while others tend to remain behind [28,82]. However, territorial defence requires a critical mass of individuals to access the benefits of the act, as captured by the volunteer's dilemma [130]. Similarly, a minimum number of participants is essential to reduce the likelihood of suffering costs. Therefore, as opposed to group hunting, the presence of impact individuals may not serve as a mechanism stabilizing collective action.

### (c) Social ties and social cohesion

Recent work on wild chimpanzee populations, combining observational and endocrinological approaches, revealed potential mechanisms by which cooperation can be maintained and collective action problems may be solved, particularly the role of group-level social cohesion. For example, the Taï chimpanzees range in larger parties [126] and show lower fission–fusion rates [39] in times of intense territoriality. Further, chimpanzee engagement in border patrols and IGEs predicts reduced male directed in-group aggression [126]. The observed cohesive in-group response probably optimizes the cost-to-benefit ratio of territorial defence, thereby facilitating collective action. Similar increased in-group cohesion was observed in a captive chimpanzee population in response to simulated out-group threats [131], even in the absence of regular territorial behaviour. In Ngogo, males were more likely to join patrols together with their maternal brothers [24] or with males with whom they also groomed more [33]. In the Taï population, male and female participation decisions in IGEs are more likely when they act together with adult maternal kin or non-kin social bond partners [32], independent of their association likelihood. Altogether, these patterns of behaviour in chimpanzees suggest that strong social relationships are determinant in successful cooperative territoriality, potentially reducing the risks of defection during IGEs [32]. Given that chimpanzees build strong social ties among unrelated individuals [107,132], these social ties constitute a factor enabling long-term collective action at the group scale. Strong social ties are associated with a stable interaction history between partners that enables individuals to benefit one another over time in a more predictable way (to support reciprocity). For example, chimpanzee meat sharing is more likely between mutually preferred grooming partners [133], and grooming can be exchanged for agonistic support [134]. Increased interaction predictability of support between bonded partners can decrease defection during collective actions by synergistically motivating participation and increasing coordination [32]. The accumulation of bonded relationships, embedded within a social network, provides a path by which group-level cooperation occurs.

The hypothesis that direct reciprocity constitutes a mechanism enabling solving collective problems finds support in chimpanzees [32] where regular social interactions within a community cement a sense of common belonging (a common affect), enabling group-level collective action and avoidance of defection. In smaller hunter–gatherer societies, repeated interaction histories between community members play a predictive role in participation in-group cooperation [135], and in some societies cooperation networks among men strongly rely on kinship and reciprocity [136]. This suggests that some group-level cooperation in humans—if only at a small scale—could be explained following the PCM. While small-scale group cooperation in chimpanzees and some group cooperative acts in humans seem to follow similar mechanisms in the form of social ties and repeated interaction histories, strong social ties and reciprocity cannot solely sustain large-scale cooperation [137]. Nonetheless, territorial defence, both on a small or larger scale, requires individuals to collectively act together with a variety of partners with whom they share differentiated types or relationships [138] (i.e. strong social ties but also those individuals that hardly interact). Therefore, how would collective action be maintained on a proximate level?

### (d) The role of oxytocin

Behavioural endocrinological studies give more insights on the physiological pathway by which reciprocity is maintained and defection potentially prevented. The oxytocinergic system is an ancient physiological system highly conserved in mammals, and involved in maternal effects and mother-offspring bonding [139]. Probably co-opted from maternal-offspring bonding and attachment the oxytocinergic system is also known to play a vital role in the formation of pair-bonds and unrelated social bond partners across taxa [25,26,140,141]. The oxytocinergic system is activated during affiliative acts in barbary macaques [142], in food sharing with in-group members in vampire bats [143], and during affiliation, post-conflict management and food sharing in chimpanzees [25,26,133,144] potentially playing an essential role in social bond maintenance and formation. In chimpanzees, oxytocin also probably plays a role in cooperative social interactions on the group level, for example, during chimpanzee searches for monkey prey and cooperative hunting [30].

The oxytocinergic system is also activated when individuals face a threat [145]. In rats, oxytocin mediates maternal defensive behaviour, with an increased secretion in response to aggression [146,147]. Oxytocin mediated protection in response to a threat is also evident in out-group competition. In humans, intranasally administrated oxytocin is known to promote in-group cooperation during intergroup Prisoner Dilemma games, by enhancing in-group cooperation and trust, and by promoting out-group defensive competition [12,148,149]. In chimpanzees, the activation of the oxytocinergic system occurs in both sexes immediately before and during IGEs and border patrols [39], and acting with kin or social bond partners during IGEs buffers stress reaction [60]. Oxytocin is involved in the mediation of the detection and avoidance of out-groups across multiple vertebrate species [145]. A review across vertebrates [48] shows that mammalian oxytocin and its analogues in birds and fishes elicit aggression and participation in out-group conflict when a threat by competitive groups is perceived, showing that this pathway is either highly conserved or at least independently activated in the same contexts. Given the effects of this neuropeptide (and others such as arginine-vasopressin) on social interactions on one side, and on social perception [150,151] on the other, the oxytocinergic system offers a physiological pathway maintaining parochial cooperation (figure 1) [12]. This physiological pathway probably acts by promoting pro-social behaviour and thus increasing in-group interests while, in parallel, increasing awareness of potential threat from the out-group, thereby increasing out-group hostility [48].

It has also been postulated that the oxytocinergic system is a potential modulator of human large-scale cooperation, as it does not invariably facilitate just cooperation, but also produces protective responses [152]. One possible explanation for the role of the oxytocinergic system in facilitating large-scale cooperation is that at some point during human evolution there was functional expansion of the system—triggering cooperation in response to an outside threat even among unfamiliar individuals. Functional expansions of the oxytocinergic system have potentially happened several times in the evolution of vertebrates [153], like being co-opted from the regulation of parturition, lactation, and mother-infant bonds, to regulating pair bond formation and social-bonds, etc. Therefore, is the additional suggested expansion of the oxytocinergic system to facilitate large-scale cooperation a unique human trait?

Findings from Triki *et al*. [48] reveal independent mobilization of oxytocin in several groups of vertebrates when facing an out-group threat, and chimpanzee in-group cooperation during an out-group threat involves oxytocin excretion [39]. The latter study also showed that the activation of the oxytocinergic system occurred as an anticipatory response to the collective group defence, potentially facilitating the essential in-group coordination and cooperation and preventing defection during out-group conflicts. While these results remain to be tested in other chimpanzee populations and species, a link between the oxytocinergic system and parochial cooperation may already exist in chimpanzees. Therefore, the pathway observed in chimpanzees may offer a transitional step upon which the oxytocinergic system can be co-opted to support not only cooperation amongst familiar group members but as well the large-scale of human cooperation.

## 6. Bonobos, an essential phylogenetic puzzle piece to revealing the evolutionary origins of human parochial altruism

The evolutionary roots of human parochial altruism can be better understood by comparing both our closest living relatives, chimpanzees and bonobos (*P. paniscus*), to humans. In both species, social bonds can support cooperation. In addition, in both species, the oxytocinergic system is involved in cooperative actions and social perception. Bonobos, like chimpanzees, live in multi-male multi-female social communities, characterized by fission–fusion and male philopatry [154]. Differences between bonobos and chimpanzees in socio-ecological conditions, such as defensibility of resources and competitive ability differential, are thought to underlie important differences in intensity of intergroup competition and intergroup relationships [155]. Bonobos are often considered less xenophobic and more tolerant than chimpanzees, as IGEs are not known to escalate into lethal outcomes [53] and as they share large overlapping home-ranges [156,157]. Intergroup relationships in bonobos are therefore considered largely peaceful [158–160]. However, IGEs in bonobos nevertheless involve increased aggression among males and females, suggesting increase in competition during these encounters [161]. As for chimpanzees [60], IGEs in bonobos are stressful events with increased cortisol levels during IGEs [161]. In Wamba, intergroup competition in bonobos involves increased cooperation among in-group males to attack out-group males and reduced in-group aggression during intergroup interactions [162], although such male coalitions are rare they resemble patterns observed in male chimpanzees. However, in Kokolopori, despite increased intergroup aggression by male bonobos during IGEs, coalitionary attacks by males are rare and testosterone levels do not increase during IGEs [163]. By contrast, intergroup interactions in Lomako and LuiKotale are considered rare [164]. These findings suggest that some degree of out-group competition is evident in bonobos, albeit to a much lesser degree than chimpanzees, and that cross-population variation in intergroup hostility occurs in bonobos. Alongside aggressive behaviour towards the out-group, bonobos can also be tolerant towards out-group members. Bonobos often engage in peaceful interactions with out-group individuals [163], female bonobos form between-group coalitions against a common target [162] and bonobos share food with out-group members [157,158]. It is suggested that bonobo intergroup competition is less intense than in chimpanzees owing to a reduction in the odds of power imbalance as a result of large and stable mixed-sex association parties [70], owing to the preponderant dominance place of females within this species [165], and potentially lower interspecific competition over resources in bonobos compared with chimpanzees [166]. This lower intensity of intergroup competition in bonobos, and thus a lower threat level as compared to chimpanzees, may explain why bonobos border patrols have not been systematically documented [167,168].

Maintaining social relationships that could enable group-level cooperative action among unrelated individuals also occurs in bonobos. Both bonobos and chimpanzees maintain in-group social ties via dyadic [25,169–171] and polyadic grooming [172,173], food sharing [26,174] and coalition formation [162,170,175]. The repeated interactions between in-group individuals probably serve as a basis upon which the mechanism of direct reciprocity can play a role in shaping non-kin cooperation, on both a dyadic and group level. This would include group-level territorial defence and group hunting in chimpanzees, but also the formation of bonobo female–female coalitions during intrasexual [176] and intergroup conflicts [162], and increased bonobo males' cohesion during IGEs [162]. Nonetheless, when directly comparing the cooperation capacities between wild chimpanzees and bonobos, experimental studies of a predator defence task show that chimpanzees cooperation performance exceeds that of bonobos [177] (but see studies in captivity that show the opposite [178]). To reveal whether variation in out-group pressure translates into greater cooperative capacities requires comparative research effort on several populations of bonobos and chimpanzees.

Research on the bonobo oxytocinergic system also reveals interesting insights on its implication in sociality and parochialism. In bonobos, captive experiments where oxytocin is intranasally administrated demonstrate that oxytocin promotes pro-social behaviour, with increased levels of grooming [179]. In wild bonobos, non-invasive endocrinological analyses [180] showed increases in urinary oxytocin following female–female sexual interactions, but not after inter-sexual interactions, and females that had more sexual interactions were more likely to engage in coalitionary aggression. Female coalitions often occur during IGEs in bonobos [162], hereby underscoring the potential proximate role of the oxytocinergic system in supporting bonobo non-kin cooperation. Given that females are thought to take an active role in maintaining tolerance during bonobo IGEs, investigating the sex-specific oxytocinergic system activity during IGEs in bonobos is an important next step for understanding the involvement of oxytocin in parochialism. That bonobo males act more aggressively during IGEs, while females form coalitions against males points towards potential different hostility strategies between sexes. Inter-sexual differences in parochialism have also been found in human studies [9], with males being more inclined to favour the in-group than females. It is possible that within-group dominance of female bonobos over males and reduced intergroup feeding competition in bonobos [156] compared with chimpanzees are responsible for reduced out-group hostility in female bonobos. More systematic studies on apes’ inter-sexual differences in the propensity for out-group hostility are thus necessary.

## 7. Alternatives and conclusion

While we have so far emphasized the pathways by which out-group conflicts may have triggered to co-evolution of non-kin group-level cooperation and out-group hostility, other hypotheses exist regarding the evolution of non-kin cooperation, especially in humans. Leading hypotheses in human evolution centre around the role of reliance on collaborative foraging and gathering or cooperative breeding as selective forces for non-kin cooperation [6,7]. Chimpanzees and bonobos are not collaborative breeders, but all populations of wild chimpanzees and bonobos hunt and share animal prey. However, although some chimpanzee populations usually hunt cooperatively [41,181], chimpanzees do access prey as single hunters or catch prey opportunistically [41,181]. In bonobos, hunting is mainly an opportunistic activity [157,182]. Further, if the hunting of animal prey would constitute a relevant selective pressure, one would expect that hunting occurs during times of necessity (e.g. when food availability is low). However, collective hunting in wild chimpanzees typically occurs during periods of high food availability [28,183] or do not show a particular seasonality [181]. Therefore, it seems likely that collaborative foraging in chimpanzees is facultative rather than a necessity. Rather than being the ultimate selective pressure on the emergence of non-kin cooperation, collective hunting and meat sharing may play a role in the reinforcement and maintenance of social bonds [26,133,184], which in turn support delayed reciprocity and group-level cooperation. To further reveal the connection between collective hunting and territorial behaviour, comparative studies across chimpanzee and bonobos populations are needed.

Nonetheless, several lines of evidence point to the role of out-group conflict as a strong selective pressure across animal taxa: first, the fitness consequences of out-group threat are evident across taxa [13,121] and can potentially be mitigated by in-group collective response. Second, there is growing evidence that increased out-group threat is linked with in-group cooperation and cohesion across taxa [14], independently of whether collective hunting [185] or cooperative breeding [126,186] is present in a taxa. Whether the causal link between out-group threat and cooperation is an example of convergent evolution or a homology remains to be explored via phylogenetic analyses. Such phylogenetic comparisons could also give insights on the ultimate mechanisms behind solving collective action problems. Further, phylogenetic investigations on the relationship between oxytocinergic system, cooperation and out-group conflicts would also help in unravelling where convergent evolution and where evolutionary homologies can be found. Whether parochial cooperation in chimpanzees constitutes the evolutionary premises of parochial altruism in humans or whether these are two convergent phenomena may never be answered. However, given that our other closest living relative, the bonobo, show both reduced out-group threat and reduced tendency for group-level cooperation, a parsimonious conclusion would be that the roots of human parochial altruism existed in the last common ancestor between chimpanzees and humans and were lost in the bonobo.

Throughout this review of the relationship between out-group conflicts, cooperative patterns, social ties, and its physiological pathway in chimpanzees, we bring forward evidence suggesting that the evolutionary roots of parochial altruism exist in humans' closest living relatives, chimpanzees. We have formulated the PCM (figure 1) as a pathway towards parochial cooperation in closed and small societies. The central factor in the model is the formation and maintenance of social ties between both kin and non-kin group members, facilitated by the oxytocinergic system, which provides the basis for parochial cooperation taking place. To reveal the evolutionary foundations of parochial cooperation and whether convergent evolution took place, we advocate that our suggested model and its components should be investigated across species that display varying levels of non-kin cooperation and intergroup competition.

## Data Availability

This article has no additional data.
